# Cinnamic Aldehyde, the main monomer component of *Cinnamon*, exhibits anti‐inflammatory property in OA synovial fibroblasts via TLR4/MyD88 pathway

**DOI:** 10.1111/jcmm.17148

**Published:** 2021-12-28

**Authors:** Pu Chen, Jun Zhou, Anmin Ruan, Lingfeng Zeng, Jun Liu, Qingfu Wang

**Affiliations:** ^1^ Department of Orthopaedic Surgery Guangdong Provincial Hospital of Traditional Chinese Medicine Guangzhou University of Chinese Medicine (The 2nd Affiliated Hospital) Guangzhou China; ^2^ Department of Orthopaedic Surgery Beijing University of Chinese Medicine Third Affiliated Hospital Beijing China; ^3^ Department of Orthopaedic Surgery Beijing Longfu Hospital Beijing China; ^4^ Bone and Joint Research Team of Degeneration and Injury Guangdong Provincial Academy of Chinese Medical Sciences Guangzhou China; ^5^ Guangdong Second Traditional Chinese Medicine Hospital (Guangdong Province Engineering Technology Research Institute of Traditional Chinese Medicine) Guangzhou China

**Keywords:** bioinformatics analysis, cinnamic aldehyde, experimental pharmacology, network pharmacology, osteoarthritis, synovial inflammation, TLR4/MyD88 signalling pathway

## Abstract

*Cinnamon* is a wildly used traditional Chinese herbal medicine for osteoarthritis (OA) treatment, but the underlying mechanism remains ambiguous. The purpose of this study is to explore the mechanism of cinnamic aldehyde (CA), a bioactive substance extracted from *Cinnamon*, on synovial inflammation in OA. A total of 144 CA‐OA co‐targeted genes were identified by detect databases (PubChem, HIT, TCMSP, TTD, DrugBank and GeneCards). The results of GO enrichment analysis indicated that these co‐targeted genes have participated in many biological processes including ‘inflammatory response’, ‘cellular response to lipopolysaccharide’, ‘response to drug’, ‘immune response’, ‘lipopolysaccharide‐mediated signalling pathway’, etc. KEGG pathway analysis showed these co‐targeted genes were mainly enriched in ‘Toll‐like receptor signalling pathway’, ‘TNF signalling pathway’, ‘NF‐kappa B signalling pathway’, etc. Molecular docking demonstrated that CA could successfully bind to TLR2 and TLR4. The results of in vitro experiments showed no potential toxicity of 10, 20 and 50 μM/L CA on human OA FLS, and CA can significantly inhibit the inflammation in LPS‐induced human FLS. Further experimental mechanism evidence confirmed CA can inhibited the inflammation in LPS‐induced human OA FLS via blocking the TLR4/MyD88 signalling pathway. Our results demonstrated that CA exhibited strong anti‐inflammation effect in OA FLS through blocking the activation of TLR4/MyD88 signalling pathway, suggesting its potential as a hopeful candidate for the development of novel agents for the treatment of OA.

## 1 INTRODUCTION

Osteoarthritis (OA), the most common degenerative joint disease and the leading cause of joint disability and persistent pain, affects 140 million people worldwide.^1^ OA is characterized by synovial inflammation, progressive articular cartilage damage and thickening of the subchondral bone that leads to pain, swelling, dysfunction of the joint and diminished quality of life.^2^, ^3^, ^4^ For a long time, articular cartilage degeneration was considered to play a crucial role in the progression of OA.^5^, ^6^ It was also reported that OA is an autoinflammatory disease caused by chondrocyte‐mediated inflammatory responses.^5^ With the deepening of our understanding of the course of OA, however, the role of soft tissues around the joint, such as synovial tissue and fat pads, in the pathogenesis of OA is increasingly recognized.^3^, ^7^, ^8^, ^9^ Synovial inflammation was found to be highly correlated with several OA symptoms such as stiffness and pain. Recent studies suggest that synovial inflammation precedes damage to other articular tissues in the development of radiographic OA,^10^ and the exosomes derived from OA fibroblast‐like synoviocytes (FLS) induce cartilage degeneration.^11^ FLS are the most abundant cell in synovial tissue and play a crucial role in the process of OA development.^12^, ^13^ Wang et.al.^3^ suggested that synovial tissue can affect articular cartilage degeneration through the innate immune system and affect OA progression. Therefore, effective inhibition of synovial inflammation may be the key to delaying cartilage damage and OA progression.

*Cinnamon* is a widely used traditional Chinese herbal medicine in treatment of OA. According to statistics, *Cinnamon* is one of the most commonly used traditional Chinese medicines in the treatment of OA.^14^, ^15^ Cinnamic aldehyde (CA), a critical bioactive substance extracted from *Cinnamon*, is known to have antioxidant, antidiabetic, anti‐inflammatory, antipyretic and anticancer properties.^16^, ^17^, ^18^, ^19^, ^20^ Our previous results show that CA can delay the degeneration of chondrocytes by inhibiting the NF‐kB signalling pathway.^2^ However, the effect of CA on OA synovial inflammation and its underlying mechanism are still unclear. Network pharmacology, an increasingly developed system for building disease–drug networks, can effectively and systematically study the pharmacological effects mechanism of action and safety of herbal medicines, especially traditional Chinese medicine (TCM).^21^, ^22^ In recent years, network pharmacology has also been used to predict potent therapeutic targets and regulate pathways of single natural products or their combinations in complex diseases.^23^ Therefore, in this study, we aimed to explore the mechanism underlying the effect of CA on synovial inflammation in OA using techniques from network pharmacology to experimental pharmacology.

## 2 METHODS

### 2.1 Cinnamic aldehyde‐associated gene mining

The PubChem, HIT and TCMSP databases were used to identify CA‐associated genes. PubChem (https://pubchem.ncbi.nlm.nih.gov) is the world's largest collection of freely accessible chemical information. The HIT database contains protein targets and potential therapeutic significance for supplementing FDA‐approved drugs.^24^ TCMSP^25^ is a unique systems pharmacology platform of Chinese herbal medicines that captures the relationships between drugs, targets and diseases. It includes chemicals, targets and drug–target networks as well as associated drug–target–disease networks. Microsoft Excel was used to integrate the obtained CA‐associated genes.

### 2.2 Osteoarthritis‐associated gene mining

The Therapeutic Target Database (TTD), DrugBank and GeneCards database were used to perform OA‐associated gene mining. The Therapeutic Target Database (TTD)^26^ is a database that provides information about known and explored therapeutic protein and nucleic acid targets, the targeted disease, pathway information and the corresponding drugs directed at each of these targets. DrugBank^27^ is a unique bioinformatics and cheminformatics resource that combines detailed drug data with comprehensive drug target information. GeneCards (www.genecards.org) is a gene database that integrates network resources and contains comprehensive information about all human gene annotations and predictions. Microsoft Excel was also used to integrate the obtained OA‐associated genes.

### 2.3 GO, KEGG functional enrichment analysis and Protein–protein interaction (PPI) network constructed

A Venn diagram online website (http://bioinformatics.psb.ugent.be/webtools/Venn/) was used to extract the intersection (co‐targeting genes) between OA‐related genes and CA‐related genes. These co‐targeting genes are considered to be potential target genes for CA treatment of OA. The Database for Annotation, Visualization, and Integrated Discovery (DAVID)^28^ was used to perform Kyoto Encyclopedia of Genes and Genomes (KEGG)^29^ analysis and Gene ontology (GO)^30^ functional enrichment analysis. The search tool for the retrieval of interacting genes (STRING) database^31^ was used to construct the protein–protein interaction (PPI) networks between these co‐targeting genes and then visualize in Cytoscape software (http://www.cytoscape.org/). Module analysis of these co‐targeted genes was performed to identify the closely connected module using molecular complex detection (MCODE).^32^ The criteria were set as follows: MCODE scores >5 and number of nodes >10. In addition, KEGG pathway enrichment analysis of molecular complex was also performed.

### 2.4 Molecular docking

The CA structure was downloaded from the TCMSP database; subsequently, the Open Babel GUI was used to convert the format from mol2 to PDB. The PDB format of TLR2 and TLR4 was downloaded from the RCSB database (http://www.rcsb.org). PyMOL software was used to remove the solvent and organic compounds. AutoDock tools 1.5.6 was used to prepare, run and analyse the docking simulations, and the docking results were analysed and visualized using AutoDock tools and PyMOL software.

### 2.5 Human osteoarthritis fibroblast‐like synoviocytes isolation and culture

The study was conducted according to the Declaration of Helsinki, and this study was approved by the Medical Ethics Committee of Beijing University of Chinese Medicine Third Affiliated Hospital. A total of 8 synovial tissue samples was obtained from patients who underwent knee arthroscopy or total knee replacement at the Beijing University of Chinese Medicine Third Affiliated Hospital. (The details of human synovial tissue donor patients are shown in Table S1). Briefly, the obtained synovial tissue obtained was immediately placed into PBS (Solarbio) containing antibiotic mixture (Penicillin and Streptomycin, Invitrogen/Thermo‐Fisher Scientific), inserted into the ice box and quickly transferred to ultra‐clean table. The samples were washed thrice in PBS, which included antibiotic mixture, and then diced into 1 × 1 × 1 mm^3^ pieces. These synovial tissue pieces were digested with 0.25% trypsin‐EDTA solution (Solarbio) for 30 min, digested in 0.2% type II collagenase (Solarbio) for 6 h, centrifuged at 176 *g* for 5 min and the supernatant was discarded. FLS were resuspended in DMEM‐H (Hyclone) with 10% FBS (ABW) and 1% antibiotic mixture. The FLS were plated at a density of 1 × 10^5^ cells/ml and incubated at 37°C in a humidified 5% CO_2_ atmosphere. Only passages 2–6 were used to avoid phenotype loss.

### 2.6 CCK‐8 assays

The cytotoxicity of CA (Sigma‐Aldrich) in human OA fibroblast‐like synoviocytes (FLS) was evaluated using the CCK‐8 assay according to the manufacturer's instructions. Human OA FLS were cultured in 96‐well plates at a density of 8 × 10^3^ cells per well for 24 h and then with different concentrations (0, 10, 20, 50 and 100 μmol/L) of CA for 24 h. Next, 10 μl CCK‐8 (Dojindo Molecular Technologies Inc) was added to each well and incubated for 4 h. The optical density was read at a wavelength of 450 nm using a microplate reader. CCK‐8 was also used to evaluate the anti‐proliferation of CA on FLS. After 4 h of starvation, the FLS were pre‐incubated with various concentrations of CA (10, 20, 50 μM) for 6 h and then stimulated or not stimulated with LPS (Bioruler) for 24 h at 37°C. The control group referred to the group in which the FLS were only treated with DMSO.

### 2.7 TLR4 overexpression

Human OA FLS were transiently transfected with a plasmid (TSINGKE) or an empty vector for 24 h using lipofectamine 2000 following the manufacturer's instructions. The FLS were pretreated with different concentrations of CA for 24 h and then stimulated with LPS (1 μg/ml) for 24 h. The mRNA and protein were collected for following experiments.

### 2.8 Quantitative real‐time PCR analysis

Quantitative real‐time PCR analysis (QPCR) was performed as described previously.^2^ Total cellular RNA was extracted from human OA FLS using TRIzol reagent (Invitrogen), and RNA was quantitated by spectrophotometry at 260 nm using an ultraviolet spectrophotometer (Thermo Scientific NanoDrop 2000). Then cDNA synthesis was performed using HiScript^®^ II Reverse Transcriptase kit (Vazyme Biotech Co). Quantitative real‐time PCR (qPCR) was performed using AceQ^®^ Universal SYBR^®^ qPCR Master Mix (Vazyme Biotech Co). The protocol of real‐time PCR was as follows: 10 min 95°C, followed by 40 cycles of 15 s 95°C and 1 min 60°C. The primer's sequences of the targeted genes are listed in Table 1. And data were analysed using 2^−ΔΔCT^ method.

**TABLE 1:** The primer's sequences of the targeted genes

Primers	Sequences
human‐IL6‐F	ACTCACCTCTTCAGAACGAATTG
human‐IL6‐R	CCATCTTTGGAAGGTTCAGGTTG
human‐IL1β‐F	CTGTCCTGCGTGTTGAAAGA
human‐IL1β‐R	TTGGGTAATTTTTGGGATCTACA
human‐TNF‐α‐F	CCTCTCTCTAATCAGCCCTCTG
human‐TNF‐α‐R	GAGGACCTGGGAGTAGATGAG
human‐β‐Actin‐F	GATTCCTATGTGGGCGACGA
human‐β‐Actin‐R	AGGTCTCAAACATGATCTGGGT

### 2.9 Western Blot analysis

As described previously,^2^, ^7^ cell lysates were prepared from FLS with RIPA lysis buffer kit (Beyotime Biotechnology), and the protein concentrations were quantified using a BCA protein assay kit (Thermo Scientific). Next, 8%–15% SDS‐PAGE gels were used to separate the cell lysates, and the samples were transferred to polyvinylidene fluoride (PVDF) membranes (Millipore). Subsequently, the membranes were incubated with primary antibodies anti‐IL‐1β, IL‐6, TNF‐α, TLR4, MyD88 (Abcam) and β‐actin (Proteintech) overnight at 4°C. After washing thrice with TBST, the membranes were incubated with Horseradish Peroxidase (HRP)‐labelled secondary antibodies (Proteintech) for 1 h at room temperature. Images were developed after reaction with a high‐sensitivity chemiluminescence reagent (Proteintech).

### 2.10 Statistical analysis

All the results were represented as the median ± standard deviation (SD). All analyses were performed using GraphPad prism 5.0 software. Two different groups were compared by independent‐sample t test, and multiple group comparisons were performed by one‐way analysis of variance (ANOVA) analysis. *p* < 0.05 was considered statistically significant. All experiments were performed at least three times independently.

## 3 RESULTS

### 3.1 Identifying CA‐OA co‐targeted genes

**FIGURE 1:**
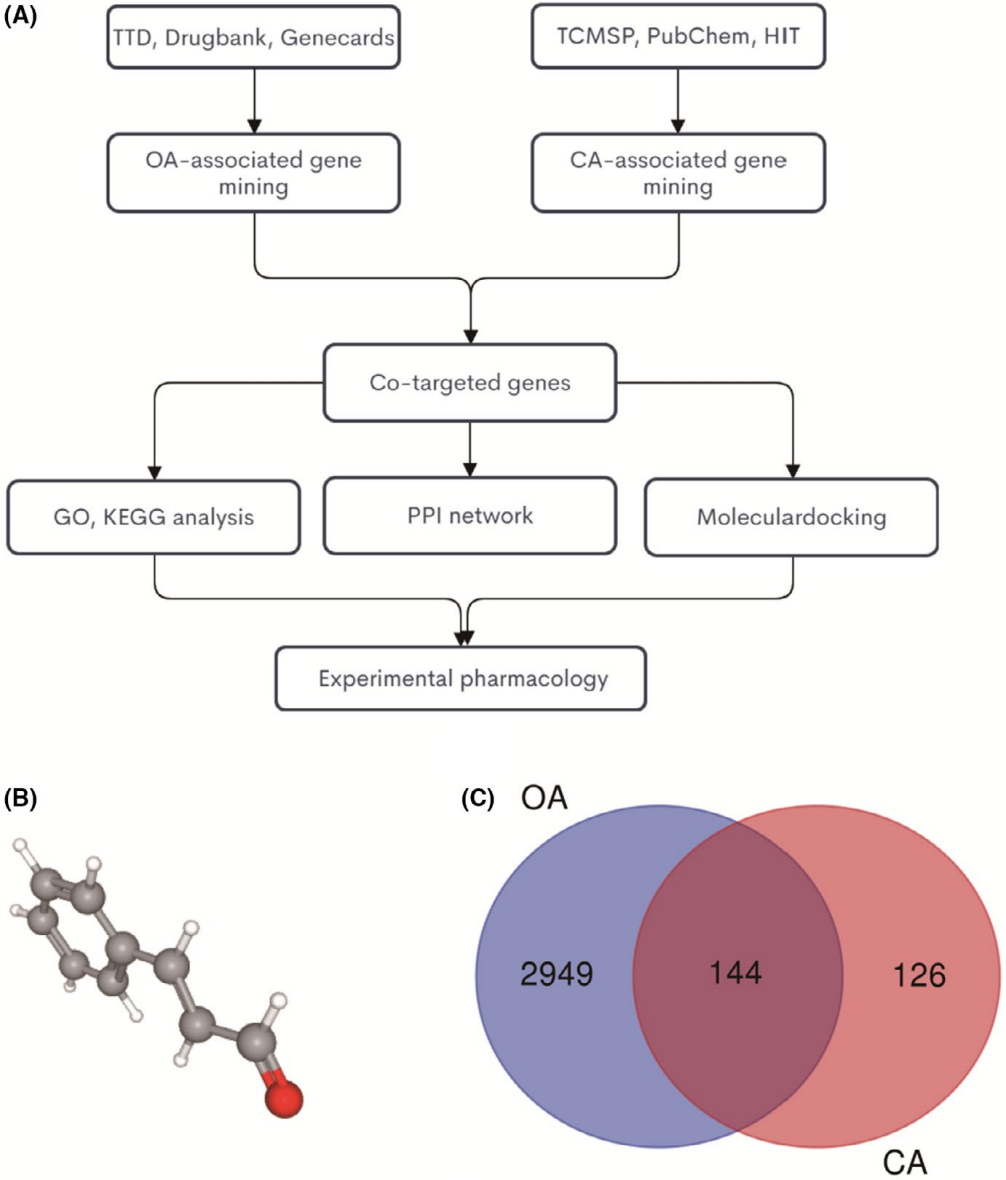
Identifying CA‐OA co‐targeted genes. (A) the flowchart of this whole analysis for this study; (B) the chemical structure of CA; (C) the Venn diagram of CA targets and OA targets genes; blue, OA‐targeted genes; red, CA‐targeted genes

The study flowchart is presented in Figure 1A, 3093 OA‐targeted genes were identified with TTD, DrugBank and Genecards databases, and 270 CA‐targeted genes were identified with TCMSP, PubChem and HIT databases. The 3D structure of CA was download in PubChem database (Figure 1B). The Venn diagram results showed that a total of 144 co‐targeted genes were identified, which are considered potential therapeutic target genes for CA in OA treatment (Figure 1C). The details of these co‐targeted genes are list in Data S1.

### 3.2 GO and KEGG pathway enrichment analysis of co‐targeted genes

**FIGURE 2:**
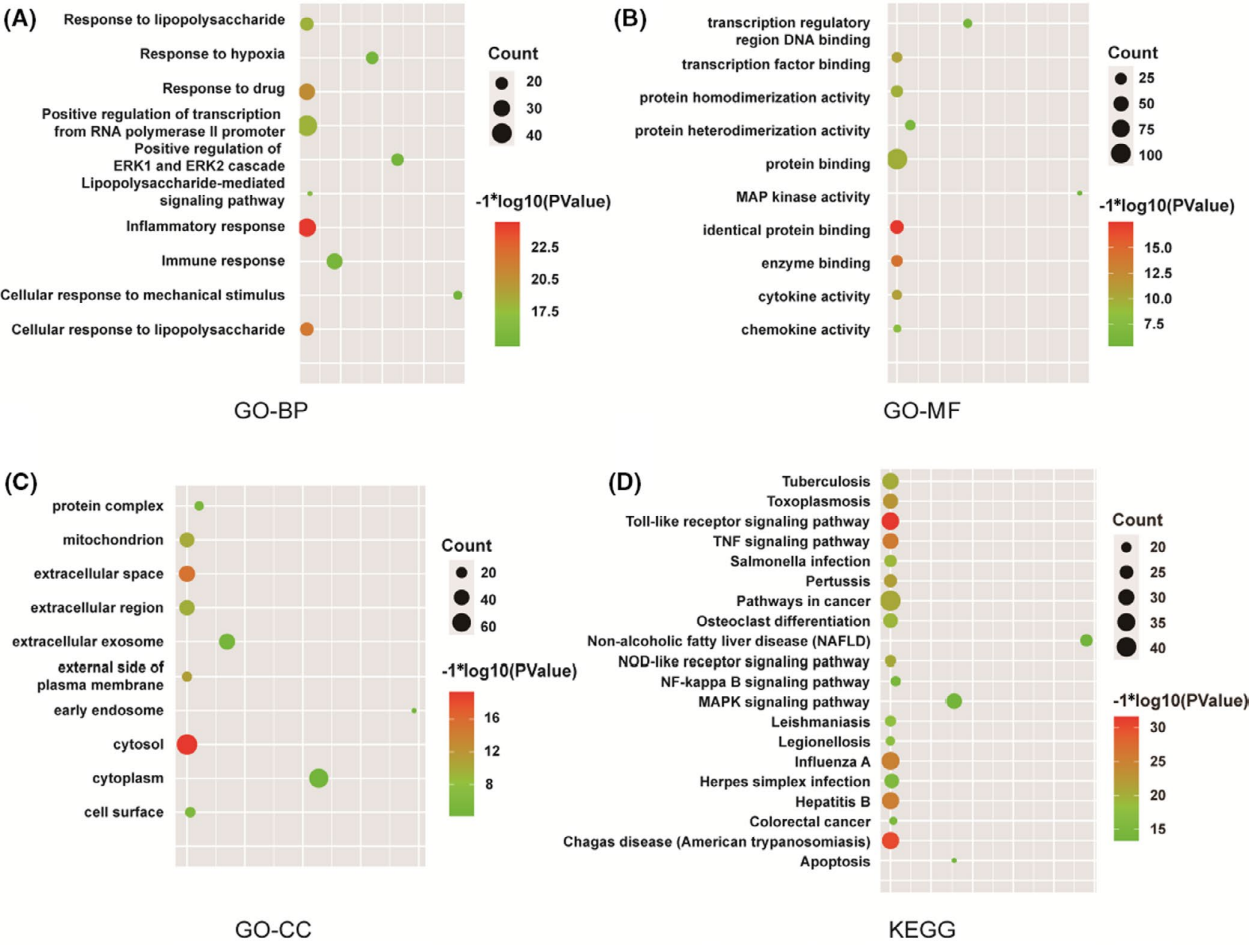
GO and KEGG pathway enrichment analysis of CA‐OA co‐targeted genes. (A) GO‐BP, GO biological processes analysis; (B) GO‐MF, GO molecular function analysis; (C) GO‐CC, GO cellular component analysis; (D) KEGG pathway enrichment analysis

The GO and KEGG pathway analyses were performed to holistically understand the functions of potential therapeutic target genes of CA in OA. The top 10 terms of GO enrichment analysis, including molecular functions (MF), biological processes (BP) and cellular components (CC), are shown in Figure 2A‐C. The results of indicated that these co‐targeted genes participate in many biological processes including ‘inflammatory response’, ‘cellular response to lipopolysaccharide’, ‘response to drug’, ‘immune response’, ‘lipopolysaccharide‐mediated signalling pathway’, etc.; have various molecular functions including ‘identical protein binding’, ‘protein binding’, ‘enzyme binding’ and ‘cytokine activity’; and mainly exert biological functions in ‘cytosol’, ‘extracellular space’ and ‘external side of plasma membrane’. Detailed information on the GO enrichment analysis is provided in Data [Link], [Link], [Link]. The top 20 terms of KEGG pathway enrichment analysis are shown in Figure 2D, including ‘Toll‐like receptor signalling pathway’, ‘Chagas disease (American trypanosomiasis)’, ‘TNF signalling pathway’, ‘NF‐kappa B signalling pathway’, etc. Detailed information on the KEGG pathway analysis is provided in Data S5.

### 3.3 Protein–protein interaction network constriction and Module analysis

**FIGURE 3:**
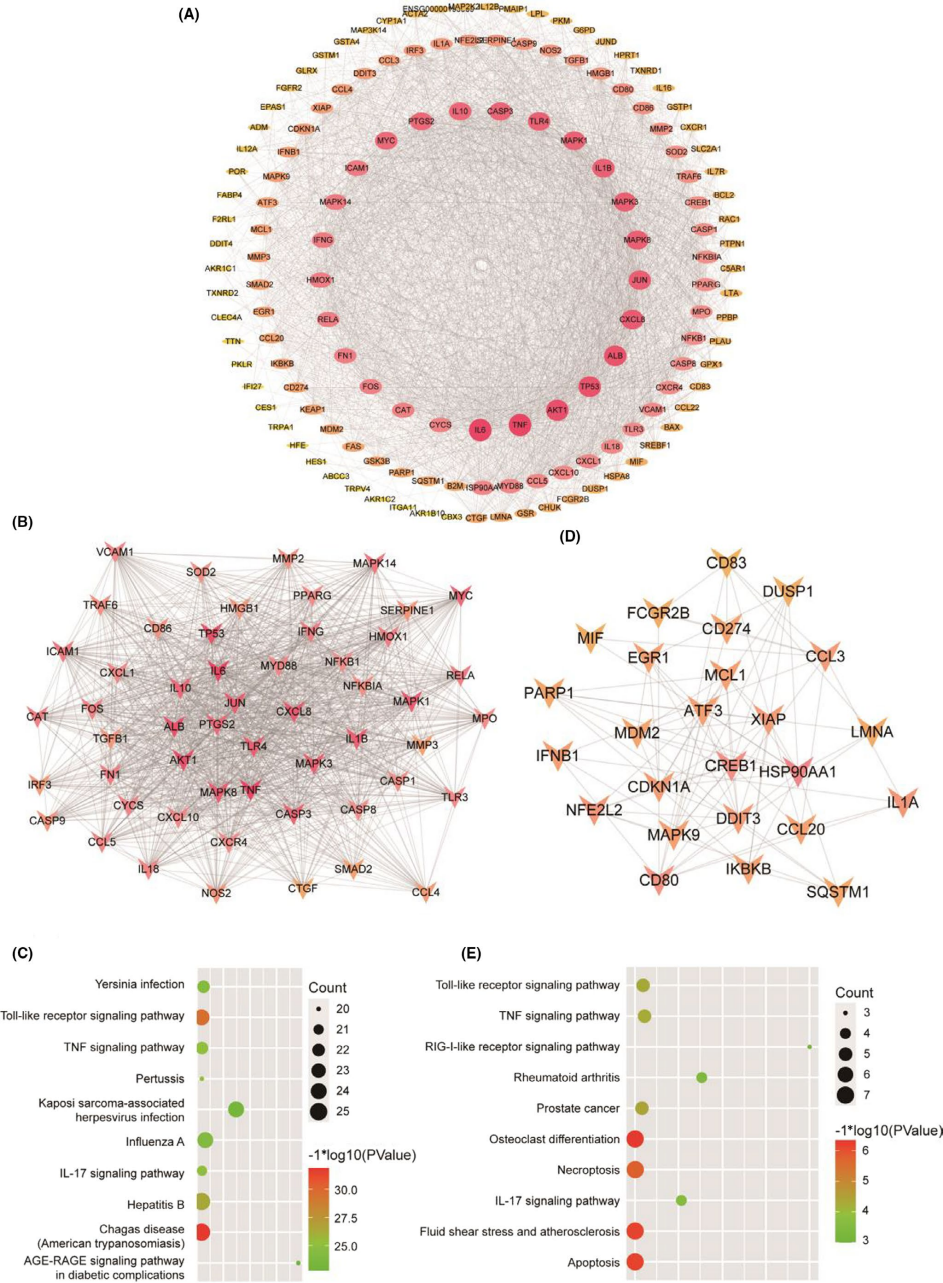
Protein–protein interaction (PPI) network constriction and module analysis. (A) PPI network of these co‐targeted genes was constructed and visualized by Cytoscape software. Red indicates the gene of higher degree, and yellow indicates the gene of lower degree. Module analysis of these co‐targeted genes when MCODE scores > 5 and number of nodes>10, (B, D) and then further analysed for KEGG pathway enrichment analysis (C, E)

The PPI network of these co‐targeted genes is shown in Figure 3A; genes with higher degree have redder colours, while the lower the yellow. The top 3 hub genes were IL6, TNF and AKT1, indicating that these genes may be the target genes for the CA treatment of OA. Two modules were identified in these co‐targeted genes with MCODE scores >5 and number of nodes >10. The first module (MCODE score = 42.308) included 53 nodes and 1100 edges, and the KEGG pathway analysis results revealed that these co‐targeted genes in modules were significantly enrichment in ‘Toll‐like receptor signalling pathway’, ‘TNF signalling pathway’, ‘IL‐17 signalling pathway’, etc. (Figure 3B,C). The second module (MCODE score = 7.250) included 25 nodes and 87 edges. As is shown in Figure 3D,E, the KEGG pathway analysis of this module suggested that ‘toll‐like receptor signalling pathway’, ‘TNF signalling pathway’, ‘osteoclast differentiation’, etc., were significantly regulated by CA in the prevention of OA. Detailed information on these two modules is presented in Data [Link], [Link], [Link].

**FIGURE 4:**
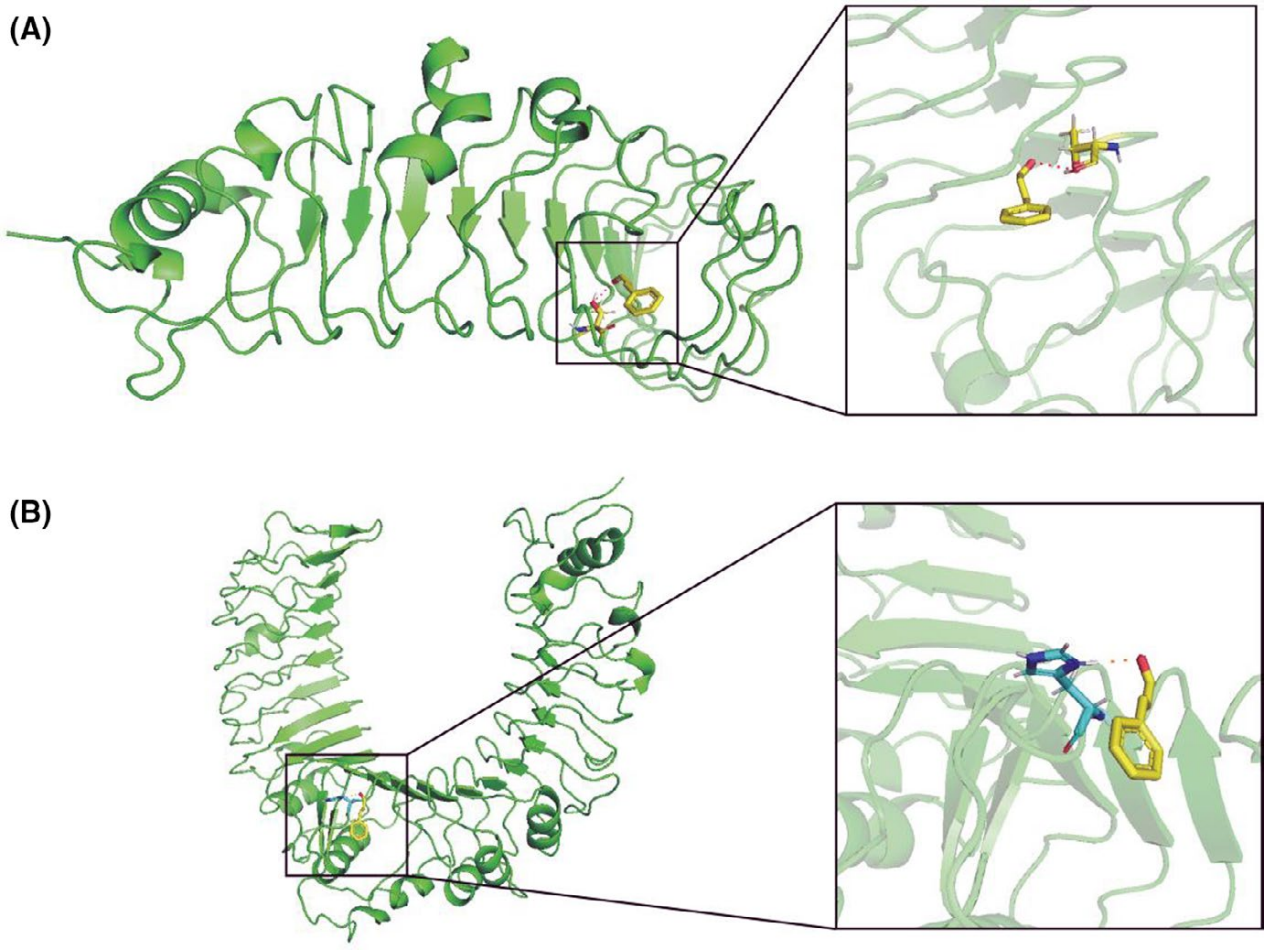
Molecular docking. Molecular docking technology was used to simulate the combination of CA with TLR2 and TLR4. (A) CA can bind to threonine 136 on TLR4; (B) CA can bind to histidine 318 on TLR2. The magnified details of the binding were showed in the right part of this figure

As the toll‐like receptor signalling pathway was successfully enriched in thrice KEGG analyses, the top 3 hub genes were all included in this pathway. Therefore, we use molecular docking technology to simulate the combination of CA with TLR2 and TLR4, which are the most studied TLR family members. As is shown in Figure 4 A‐B, CA can bind to threonine 136 on TLR4 and histidine 318 on TLR2. And the binding energy of CA and TLR2 is −4.04 kcal/mol, while TLR4 is −5.12 kcal/mol. Meanwhile, our previous study^3^ suggested that synovial inflammation can activate the cartilage innate immune system through the TLR4/MyD88 signalling pathway. Therefore, we hypothesized that CA can exhibit the effect of treating OA by regulating the TLR4/MyD88 signalling pathway.

### 3.4 Cinnamic aldehyde inhibits the expression of inflammatory factors in LPS‐induced human FLS

**FIGURE 5:**
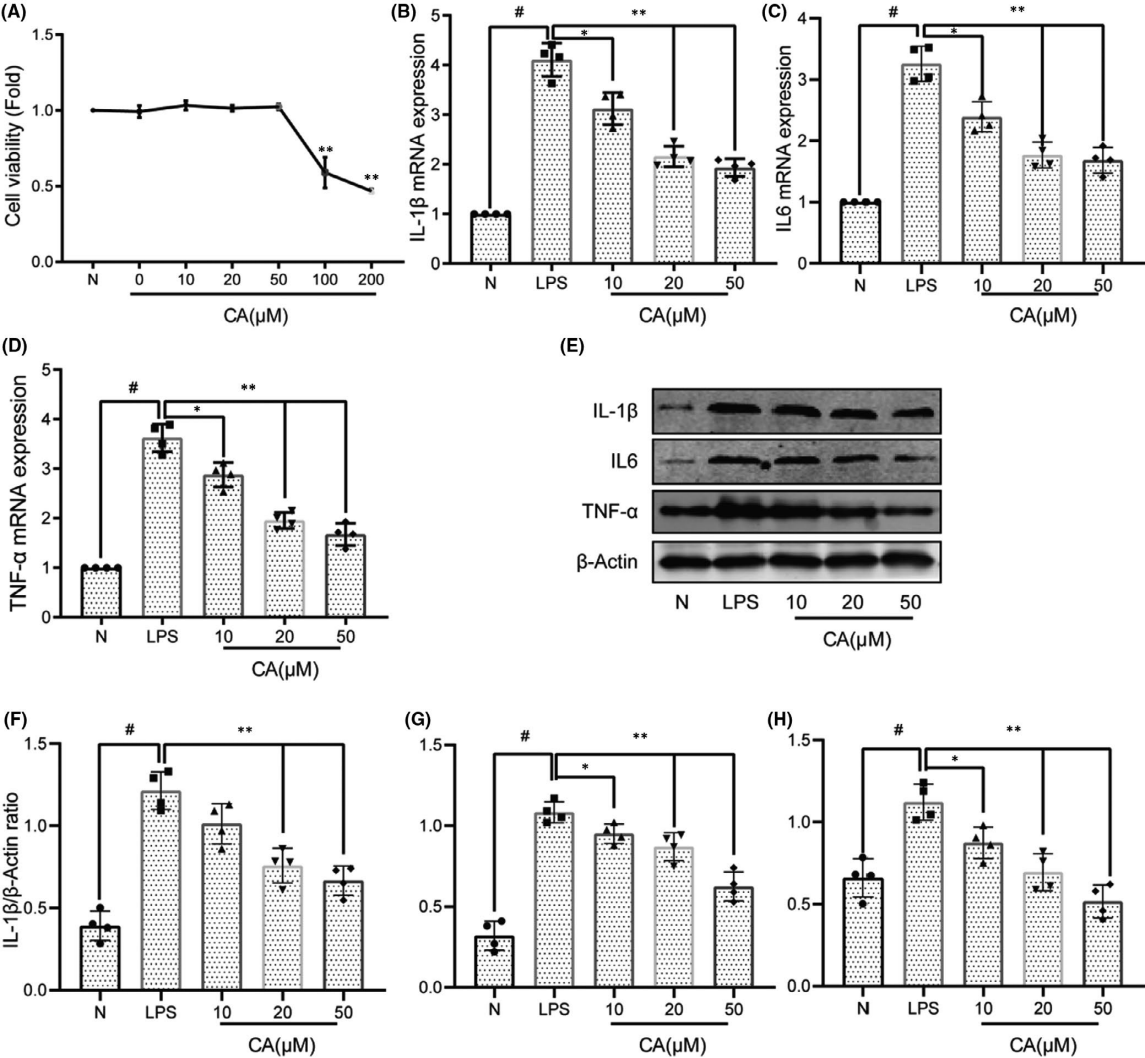
Cinnamic aldehyde (CA) inhibits the expression of inflammatory factors in LPS‐induced human fibroblast‐like synoviocytes (FLS). (A) Human osteoarthritis (OA) fibroblast‐like synoviocytes (FLS) were incubated with different concentrations of CA (0, 10, 20, 50, 100 and 200 μM) for 24 h and the cell viability was evaluated using the CCK‐8 assay; (B) FLS were pretreated with various concentrations of CA (10, 20 and 50 μM) and then stimulated or not stimulated with LPS (1 μg/ml) for 24 h. RT‐qPCR was used to assess the mRNA expression levels of IL‐1β, IL‐6 and TNF‐α (B‐D); WB was used to assess the protein expression levels of IL‐1β, IL‐6 and TNF‐α, and then quantification analysis using ImageJ software (E‐H). All experiments were performed at least 3 times independently, *p < 0.05; **p < 0.01; ^#^ *p* < 0.01

The cells were incubated with different concentrations of CA (0, 10, 20, 50, 100 and 200 μmol/L) for 24 h, and cell viability was determined using the CCK‐8 assay to evaluate the potential cytotoxicity of CA in human FLSs. As is shown in Figure 5A, CA had no cytotoxicity in human FLS at the concentrations of 10, 20 and 50 μmol/L (*p* > 0.05), which were thus selected for subsequent experiment in vitro experiments.

To evaluate the effect of CA on OA synovial inflammation, the inhibition of CA on inflammatory factors was firstly detected in LPS‐induced FLS. The FLS were treated with different concentrations of CA (10, 20, 50 μmol/L) and then stimulated or not stimulated with LPS (1 μg/ml) for 24 h, followed by measurement of IL‐1β, IL‐6 and TNF‐α levels using PCR and WB assays. As is shown in Figure 5B‐H, the levels of IL‐1β, IL‐6 and TNF‐α were significantly increased after stimulated with LPS (*p* < 0.01). However, pre‐treatment with different concentrations of CA (20 and 50 μmol/L) significantly inhibited the expression of IL‐1β, IL‐6 and TNF‐α in LPS‐induced human FLS (*p* < 0.05), with a dose‐dependent manner.

### 3.5 Cinnamic aldehyde inhibits the inflammation in LPS‐induced human OA FLS via blocking the TLR4/MyD88 signalling pathway

**FIGURE 6:**
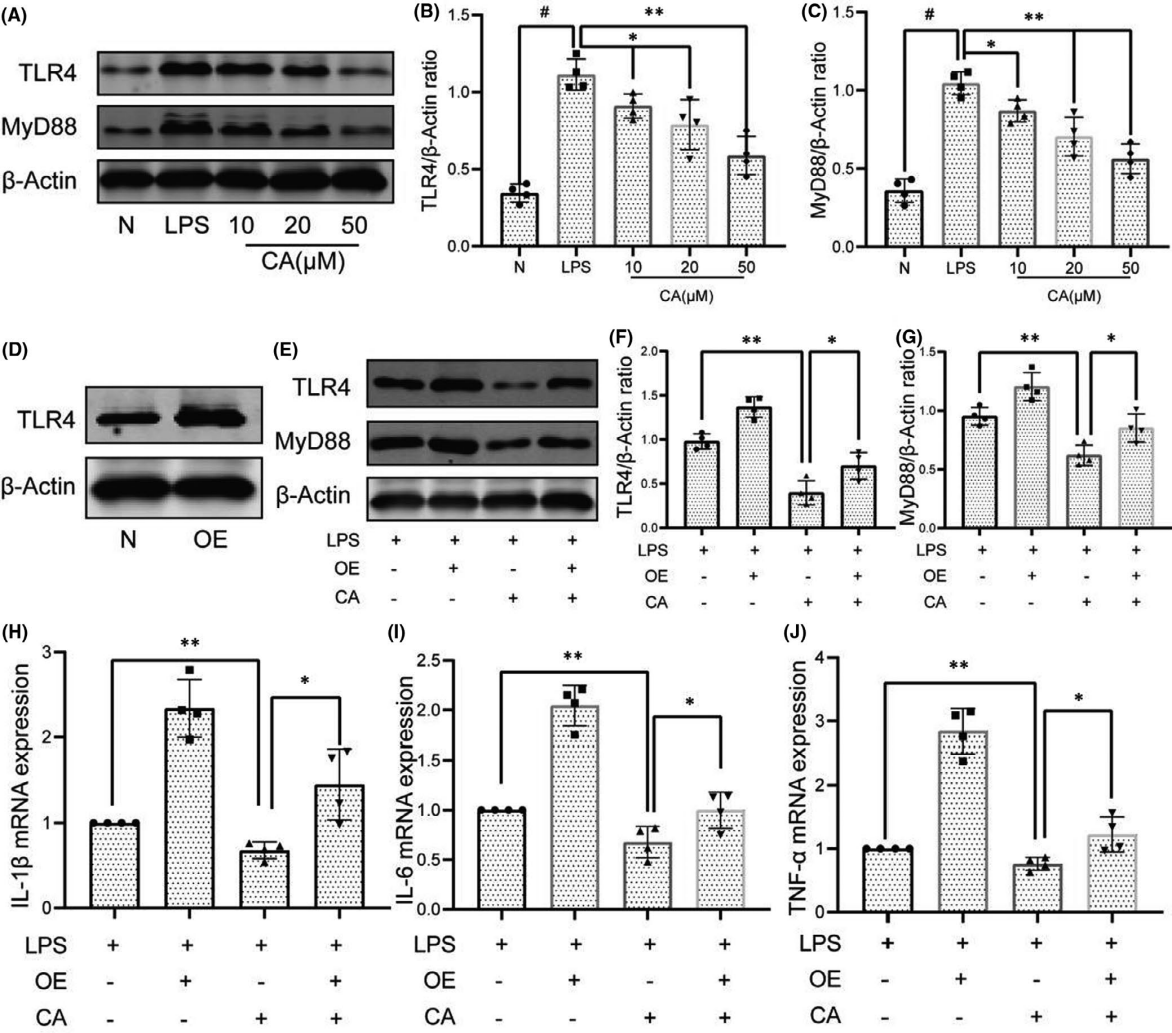
Cinnamic aldehyde (CA) inhibits the inflammation in LPS‐induced human osteoarthritis (OA) fibroblast‐like synoviocytes (FLS) via blocking the TLR4/MyD88 signalling pathway. Human OA FLS were pretreated for 24 h with different concentrations of CA (10, 20 and 50 μM) and then stimulated or not stimulated with LPS for 24 h. The protein expression levels of TLR4 and MyD88 were determined using Western blot assay (A) and then quantified (B‐C). (D) Treatment with TLR4 plasmid significantly upregulated the expression of TLR4. (E‐G) Pretreatment with CA inhibited the activation of TLR4/MyD88 signalling pathway in LPS‐induced human OA FLS. (H‐J) RT‐qPCR was used to assess the mRNA expression levels of IL‐1β, IL‐6 and TNF‐α. OE, overexpression, FLS treatment with TLR4 plasmid. All experiments were performed at least 3 times independently. *p < 0.05; **p < 0.01; ^#^ *p* < 0.01

Our previous study^3^ suggested that synovial inflammation can activate the cartilage innate immune system through the TLR4/MyD88 signalling pathway, thereby affecting the progression of OA. And the CA and TLR4 had better binding energy than TLR2. Therefore, we investigated the expression of TLR4 and MyD88 in LPS‐induced FLS. As is shown in Figure 6A‐C, the LPS‐induced FLS showed significantly upregulated TLR4 and MyD88, whereas pre‐treatment with CA led to the downregulation the expression of TLR4 and MyD88, indicating that CA can inhibited the activation of TLR4/MyD88 signalling pathway in LPS‐induced FLS. Furthermore, to demonstrate the necessity of the TLR4/MyD88 signalling pathway in CA‐mediated inhibition of OA synovial inflammation, FLS were stimulation with the TLR4 plasmid (Figure 6D). As presented in Figure 6E‐G and Figure S1, the expression of TLR4 and MyD88 was significantly upregulated in LPS + TLR4 overexpression (OE) group, where CA (20 μM) inhibited the expression of TLR4 and MyD88 (*p* < 0.05). In addition, we evaluated the effect of overexpression of TLR4 on CA's inhibition of OA synovial inflammation. As is shown in Figure 6H‐J, CA (20 μM) significantly downregulated the expression of inflammatory cytokines (IL‐1β, IL‐6 and TNF‐α) in LPS‐induced FLS, while overexpression of TLR4 significantly reversed the inhibitory effect of CA on synovial inflammation. These results indicated that CA inhibits the OA synovial inflammation via blocking the TLR4/MyD88 signalling pathway.

## 4 DISCUSSION

Osteoarthritis is one of the most common degenerative bone and joint diseases, especially in the knee joint.^7^, ^33^ Total joint arthroplasty (TKA) is regarded as the last choice for end‐stage osteoarthritis.^34^ However, there is a risk of serious complications after TKA, such as functional dissatisfaction, infection and revision.^34^ Therefore, slowing down the progression of OA has always been the focus of orthopaedic physicians. Synovial inflammation, one of the typical pathological features of OA, plays a critical role in the development of OA.^7^ Synovial inflammation not only affects cartilage degeneration of OA but also affects the inflammation of intra‐articular fat pad.^9^, ^35^ Kato et.al.^11^ suggested that synovial inflammation can induce OA‐like changes in normal human articular chondrocytes and promote cartilage degradation. Meanwhile, Atukorala et al.^10^ performed a prospective observational cohort study that showed synovial inflammation as a precursor of radiographic OA, which further illustrates the critical role of synovial inflammation in the early and mid‐stage OA.

*Cinnamon* is widely used to treat low back pain, diabetes, cancer, cardiovascular and respiratory diseases. Pharmacological research suggested that CA, an active ingredient extracted from *Cinnamon*, can inhibit the angiogenic activity of cancer by regulating the mTOR pathway‐mediated suppression of HIF‐1α expression.^36^ CA can inhibit the TLR, JNK, p38 and NF‐κB signalling pathways, regulate autophagy and inhibit inflammation and bone loss in mouse periodontitis.^37^ Moreover, it can inhibit neointimal hyperplasia after carotid artery balloon injury and prevent hyperglycaemia‐induced endothelial dysfunction via the activation of the expression of Nrf2^38^ and can also exhibit anti‐inflammatory and anti‐oxidant effects in rheumatoid arthritis patients.^39^ However, to our knowledge, the effects of CA on OA synovial inflammation as well as the potential therapeutic mechanism are still unclear. In this study, network pharmacology, bioinformatics and experimental verification were used to clarify the mechanism underlying the effect of CA as a potential treatment for OA.

In total, 144 CA‐OA co‐targeted genes were identified in this study, including chemokines and cytokines such as ILs, CCLs, CXCLs; classic signalling pathway markers such as MAPKs, NF‐κB, TLRs; and cell phenotype biomarkers such as CASPs, BCL, BAX, etc. The results of GO‐BP suggest that these co‐targeted genes were mainly enriched in ‘inflammatory response’, ‘response to drug’, ‘immune response’, etc. And the KEGG pathway enrichment analysis suggests that these co‐targeted genes were mainly participated in ‘toll‐like receptor signalling pathway’, ‘TNF signalling pathway’, ‘NF‐kappa B signalling pathway’, etc. The role of inflammation in the course of OA is well recognized. Our previous studies have also shown that CA can inhibit OA cartilage inflammation and cartilage degeneration through the NF‐kappa B signalling pathway, which is consistent with the results of GO‐BP and KEGG enrichment analysis.^2^ Meanwhile, the role of innate immune system in the progression of OA has also received increasing attention. Some studies suggested that the innate immune system is an active participant in synovial inflammation and cartilage catabolism and can initiate a series of molecular mechanisms to repair and reverse the damage.^40^, ^41^ Two modules were identified in these co‐targeted genes, and the KEGG pathway enrichment analysis results of these two modules both included the TLR and TNF signalling pathway. Previous research suggested that synovial inflammation can affect the cartilage innate immune system through TLR4/MyD88 signalling pathway, induce cartilage inflammation and degeneration and then aggravate the OA process.^3^ And the molecular docking shown CA can bind to threonine 136 on TLR4. Therefore, we hypothesized that CA could inhibit OA synovial inflammation through the TLR4/MyD88 signalling pathway.

Firstly, we investigated the effect of CA on inflammatory cytokines (IL‐1β, IL‐6 and TNF‐α) in LPS‐induced human OA FLS and found that CA (20 and 50 μmol/L) can significantly inhibited the expression of IL‐1β, IL‐6 and TNF‐α in LPS‐induced FLS (*p* < 0.05). Meanwhile, CA also inhibited the activation of TLR4/MyD88 signalling pathway in LPS‐induced OA FLS (*p* < 0.05). Moreover, we found that when the TLR4 plasmid was transfected into FLS, CA specifically inhibited the activation of the TLR4/MyD88 signalling pathway, which further explained the necessity of the TLR4/MyD88 signalling pathway in the CA‐mediated inhibition of OA synovial inflammation. However, this article still has some limitations. This article uses the results of network pharmacology, molecular docking and cell experiments to research the potential mechanism of CA in the treatment of OA, but lacks animal experimental data. Meanwhile, in this article, 8 synovial samples were used to study the effect of CA on OA synovial inflammation. It is necessary to select larger samples to avoid the effect of selection bias, sample bias on the result.

Collectively, this study is the first to demonstrate the anti‐inflammatory effects of CA on OA synovial inflammation from the network pharmacology and bioinformatics analysis to experimental pharmacology. In LPS‐induced human OA FLS, CA significantly inhibit OA synovial inflammation via blocking the TLR4/MyD88 signalling pathway. Our study provides new insights into the investigation of the anti‐inflammatory effects of CA in OA using integrative pharmacology‐based approaches, and the results suggest that CA may be a potential therapeutic agent for OA.

## CONFLICT OF INTERESTS

The authors declare that they have no conflict of interest.

## AUTHOR CONTRIBUTIONS

**Pu Chen:** Conceptualization (equal); Data curation (equal); Methodology (equal); Validation (equal); Visualization (equal); Writing – original draft (equal); Writing – review & editing (equal). **Jun Zhou:** Data curation (equal); Methodology (equal); Resources (equal); Validation (equal); Visualization (equal). **Anmin Ruan:** Methodology (equal); Software (equal); Validation (equal); Visualization (equal). **Lingfeng Zeng:** Investigation (equal); Resources (equal); Software (equal). **Jun Liu:** Funding acquisition (equal); Project administration (equal); Supervision (equal). **QingFu Wang:** Funding acquisition (equal); Project administration (equal); Resources (equal); Supervision (equal).

## Supporting information

Figure S1

Table S1

Data S1

Data S2

Data S3

Data S4

Data S5

Data S6

Data S7

Data S8

## Data Availability

The data that support the findings of this study are available from the corresponding author upon reasonable request.
