# Supplementary material for: Cinnamic Aldehyde, the main monomer component of Cinnamon, exhibits anti‐inflammatory property in OA synovial fibroblasts via TLR4/MyD88 pathway
Source: J Cell Mol Med. 2021 Dec 28;26(3):913–24. doi: 10.1111/jcmm.17148 (PMC8817122; doi:10.1111/jcmm.17148)

**Supplementary figure 1:** Immunofluorescence results of CA inhibits synovial inflammation via blocking the TLR4.


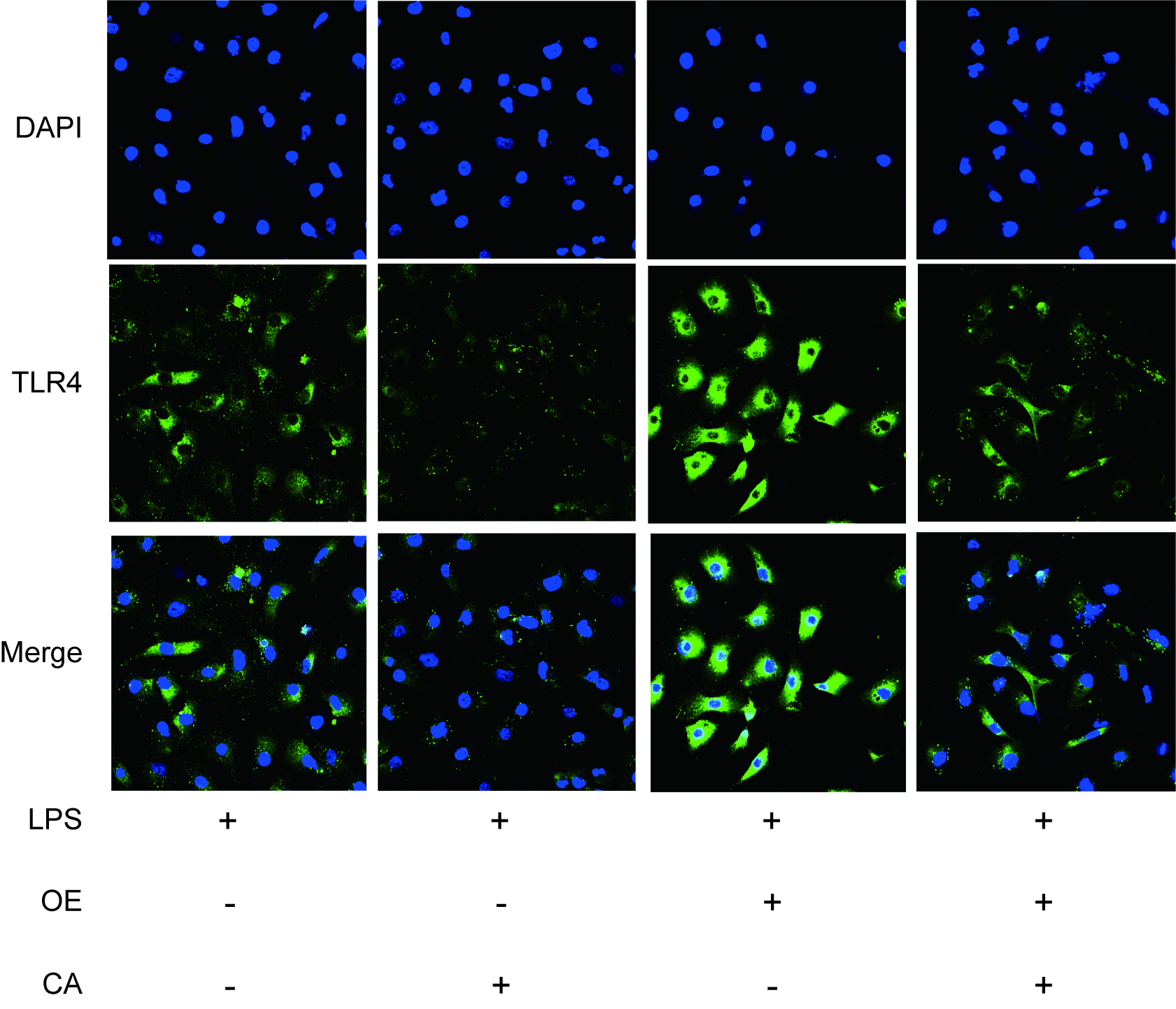

Supplement: Supplementary file 1 — Figure S1 [file JCMM-26-913-s004.docx]
