# Supplementary material for: Cinnamic Aldehyde, the main monomer component of Cinnamon, exhibits anti‐inflammatory property in OA synovial fibroblasts via TLR4/MyD88 pathway
Source: J Cell Mol Med. 2021 Dec 28;26(3):913–24. doi: 10.1111/jcmm.17148 (PMC8817122; doi:10.1111/jcmm.17148)
Supplement: Supplementary file 2 — Table S1 [file JCMM-26-913-s005.docx]

Supplementary table 1: The details of the human synovial tissue donor patients.

|  | KOA patients |
| --- | --- |
| No. | 8 |
| Male/Female | 3/5 |
| Age | 58.27±9.41 |
| BMI | 27.84±3.67 |
