# Supplementary material for: Cinnamic Aldehyde, the main monomer component of Cinnamon, exhibits anti‐inflammatory property in OA synovial fibroblasts via TLR4/MyD88 pathway
Source: J Cell Mol Med. 2021 Dec 28;26(3):913–24. doi: 10.1111/jcmm.17148 (PMC8817122; doi:10.1111/jcmm.17148)
Supplement: Supplementary file 3 — Data S1 [file JCMM-26-913-s002.docx]

Supplementary material 1: The details of these co-targeted genes

| Gene Symbol | Description | Gene ID |
| --- | --- | --- |
| ABCC3 | ATP binding cassette subfamily C member 3 | 8714 |
| ACTA2 | Actin Alpha 2, Smooth Muscle | 59 |
| ADM | Adrenomedullin | 133 |
| AKR1B10 | Aldo-keto reductase family 1 member B10 | 57016 |
| AKR1C1 | Aldo-Keto Reductase Family 1 Member C1 | 1645 |
| AKR1C2 | Aldo-keto reductase family 1 member C2 | 1646 |
| AKT1 | AKT Serine/Threonine Kinase 1 | 207 |
| ALB | Albumin | 213 |
| ATF3 | Activating Transcription Factor 3 | 467 |
| B2M | Beta-2-Microglobulin | 567 |
| BAX | BCL2 Associated X, Apoptosis Regulator | 581 |
| BCAP29 | B Cell Receptor Associated Protein 29 | 55973 |
| BCL2 | BCL2 Apoptosis Regulator | 596 |
| C5AR1 | Complement C5a Receptor 1 | 728 |
| CASP1 | Caspase 1 | 834 |
| CASP3 | Caspase 3 | 836 |
| CASP8 | Caspase 8 | 841 |
| CASP9 | Caspase 9 | 842 |
| CAT | Catalase | 847 |
| CBX3 | Chromobox 3 | 11335 |
| CCL20 | C-C Motif Chemokine Ligand 20 | 6364 |
| CCL22 | C-C Motif Chemokine Ligand 22 | 6367 |
| CCL3 | C-C Motif Chemokine Ligand 3 | 414062 |
| CCL4 | C-C Motif Chemokine Ligand 4 | 6351 |
| CCL5 | C-C Motif Chemokine Ligand 5 | 6352 |
| CCN2 | Cellular Communication Network Factor 2 | 1490 |
| CD274 | CD274 Molecule | 29126 |
| CD80 | CD80 Molecule | 941 |
| CD83 | CD83 Molecule | 9308 |
| CD86 | CD86 Molecule | 942 |
| CDKN1A | Cyclin Dependent Kinase Inhibitor 1A | 1026 |
| CES1 | Carboxylesterase 1 | 1066 |
| CHUK | Component of Inhibitor of Nuclear Factor Kappa B Kinase Complex | 1147 |
| CLEC4A | C-Type Lectin Domain Family 4 Member A | 50856 |
| CREB1 | CAMP Responsive Element Binding Protein 1 | 1385 |
| CXCL1 | C-X-C Motif Chemokine Ligand 1 | 2919 |
| CXCL10 | C-X-C Motif Chemokine Ligand 10 | 3627 |
| CXCL8 | C-X-C Motif Chemokine Ligand 8 | 3576 |
| CXCR1 | C-X-C Motif Chemokine Receptor 1 | 3577 |
| CXCR4 | C-X-C Motif Chemokine Receptor 4 | 7852 |
| CYCS | Cytochrome C, Somatic | 54205 |
| CYP1A1 | Cytochrome P450 Family 1 Subfamily A Member 1 | 1543 |
| DDIT3 | DNA Damage Inducible Transcript 3 | 1649 |
| DDIT4 | DNA Damage Inducible Transcript 4 | 54541 |
| DUSP1 | Dual Specificity Phosphatase 1 | 1843 |
| EGR1 | Early Growth Response 1 | 1958 |
| EPAS1 | Endothelial PAS Domain Protein 1 | 2034 |
| F2RL1 | F2R Like Trypsin Receptor 1 | 2150 |
| FABP4 | Fatty Acid Binding Protein 4 | 2167 |
| FAS | Fas Cell Surface Death Receptor | 355 |
| FCGR2B | Fc Fragment of IgG Receptor IIb | 2213 |
| FGFR2 | Fibroblast Growth Factor Receptor 2 | 2263 |
| FN1 | Fibronectin 1 | 2335 |
| FOS | Fos Proto-Oncogene, AP-1 Transcription Factor Subunit | 2353 |
| G6PD | Glucose-6-Phosphate Dehydrogenase | 2539 |
| GLRX | Glutaredoxin | 2745 |
| GPX1 | Glutathione Peroxidase 1 | 2876 |
| GSK3B | Glycogen Synthase Kinase 3 Beta | 2932 |
| GSR | Glutathione-Disulfide Reductase | 2936 |
| GSTA4 | Glutathione S-Transferase Alpha 4 | 2941 |
| GSTM1 | Glutathione S-Transferase Mu 1 | 2944 |
| GSTP1 | Glutathione S-Transferase Pi 1 | 2950 |
| GSTT1 | Glutathione S-Transferase Theta 1 | 2953 |
| H4C1 | H4 clustered histone 1 | 8329 |
| HES1 | Hes Family BHLH Transcription Factor 1 | 3280 |
| HFE | Homeostatic Iron Regulator | 3077 |
| HMGB1 | High Mobility Group Box 1 | 3146 |
| HMOX1 | Heme Oxygenase 1 | 3162 |
| HPRT1 | Hypoxanthine Phosphoribosyltransferase 1 | 3251 |
| HSP90AA1 | Heat Shock Protein 90 Alpha Family Class A Member 1 | 3320 |
| HSPA8 | Heat Shock Protein Family A (Hsp70) Member 8 | 3312 |
| ICAM1 | Intercellular Adhesion Molecule 1 | 3383 |
| IFI27 | Interferon Alpha Inducible Protein 27 | 3429 |
| IFNB1 | Interferon Beta 1 | 3456 |
| IFNG | Interferon Gamma | 3458 |
| IKBKB | Inhibitor Of Nuclear Factor Kappa B Kinase Subunit Beta | 3551 |
| IL10 | Interleukin 10 | 3586 |
| IL12A | Interleukin 12A | 3592 |
| IL12B | Interleukin 12B | 3593 |
| IL16 | Interleukin 16 | 3603 |
| IL18 | Interleukin 18 | 3606 |
| IL1A | Interleukin 1 Alpha | 3552 |
| IL1B | Interleukin 1 Beta | 3553 |
| IL6 | Interleukin 6 | 3569 |
| IL7R | Interleukin 7 Receptor | 3575 |
| IRF3 | Interferon Regulatory Factor 3 | 3661 |
| ITGA11 | Integrin Subunit Alpha 11 | 22801 |
| JUN | Jun Proto-Oncogene, AP-1 Transcription Factor Subunit | 3725 |
| JUND | JunD Proto-Oncogene, AP-1 Transcription Factor Subunit | 3727 |
| KEAP1 | Kelch Like ECH Associated Protein 1 | 9817 |
| LMNA | Lamin A/C | 4000 |
| LPL | Lipoprotein Lipase | 4023 |
| LTA | Lymphotoxin Alpha | 4049 |
| MAP2K2 | Mitogen-Activated Protein Kinase Kinase 2 | 5605 |
| MAP3K14 | Mitogen-Activated Protein Kinase Kinase Kinase 14 | 9020 |
| MAPK1 | Mitogen-Activated Protein Kinase 1 | 5594 |
| MAPK14 | Mitogen-Activated Protein Kinase 14 | 1432 |
| MAPK3 | Mitogen-Activated Protein Kinase 3 | 5595 |
| MAPK8 | Mitogen-Activated Protein Kinase 8 | 5599 |
| MAPK9 | Mitogen-Activated Protein Kinase 9 | 5601 |
| MCL1 | MCL1 Apoptosis Regulator, BCL2 Family Member | 4170 |
| MDM2 | MDM2 Proto-Oncogene | 4193 |
| MIF | Macrophage Migration Inhibitory Factor | 4282 |
| MMP2 | Matrix Metallopeptidase 2 | 4313 |
| MMP3 | Matrix Metallopeptidase 3 | 4314 |
| MPO | Myeloperoxidase | 4353 |
| MYC | MYC Proto-Oncogene, BHLH Transcription Factor | 4609 |
| MYD88 | MYD88 Innate Immune Signal Transduction Adaptor | 4615 |
| NFE2L2 | Nuclear Factor, Erythroid 2 Like 2 | 4780 |
| NFKB1 | Nuclear Factor Kappa B Subunit 1 | 4790 |
| NFKBIA | NFKB Inhibitor Alpha | 4792 |
| NOS2 | Nitric Oxide Synthase 2 | 4843 |
| PARP1 | Poly(ADP-Ribose) Polymerase 1 | 142 |
| PKLR | Pyruvate Kinase L/R | 5313 |
| PKM | Pyruvate Kinase M1/2 | 5315 |
| PLAU | Plasminogen Activator, Urokinase | 5328 |
| PMAIP1 | Phorbol-12-Myristate-13-Acetate-Induced Protein 1 | 5366 |
| POR | Cytochrome P450 Oxidoreductase | 5447 |
| PPARG | Peroxisome Proliferator Activated Receptor Gamma | 5468 |
| PPBP | Pro-Platelet Basic Protein | 5473 |
| PTGS2 | Prostaglandin-Endoperoxide Synthase 2 | 5743 |
| PTPN1 | Protein Tyrosine Phosphatase Non-Receptor Type 1 | 5770 |
| RAC1 | Rac Family Small GTPase 1 | 5879 |
| RELA | RELA Proto-Oncogene, NF-KB Subunit | 5970 |
| SERPINE1 | Serpin Family E Member 1 | 5054 |
| SLC2A1 | Solute Carrier Family 2 Member 1 | 6513 |
| SMAD2 | SMAD Family Member 2 | 4087 |
| SOD2 | Superoxide Dismutase 2 | 6648 |
| SQSTM1 | Sequestosome 1 | 8878 |
| SREBF1 | Sterol Regulatory Element Binding Transcription Factor 1 | 6720 |
| TGFB1 | Transforming Growth Factor Beta 1 | 7040 |
| TLR3 | Toll Like Receptor 3 | 7098 |
| TLR4 | Toll Like Receptor 4 | 7099 |
| TNF | Tumor Necrosis Factor | 7124 |
| TP53 | Tumor Protein P53 | 7157 |
| TRAF6 | TNF Receptor Associated Factor 6 | 7189 |
| TRPA1 | Transient Receptor Potential Cation Channel Subfamily A Member 1 | 8989 |
| TRPV1 | Transient Receptor Potential Cation Channel Subfamily V Member 1 | 7442 |
| TRPV4 | Transient Receptor Potential Cation Channel Subfamily V Member 4 | 59341 |
| TTN | Titin | 7273 |
| TXNRD1 | Thioredoxin Reductase 1 | 7296 |
| TXNRD2 | Thioredoxin Reductase 2 | 10587 |
| VCAM1 | Vascular Cell Adhesion Molecule 1 | 7412 |
| XIAP | X-Linked Inhibitor of Apoptosis | 331 |
