# Supplementary material for: Cinnamic Aldehyde, the main monomer component of Cinnamon, exhibits anti‐inflammatory property in OA synovial fibroblasts via TLR4/MyD88 pathway
Source: J Cell Mol Med. 2021 Dec 28;26(3):913–24. doi: 10.1111/jcmm.17148 (PMC8817122; doi:10.1111/jcmm.17148)
Supplement: Supplementary file 8 — Data S6 [file JCMM-26-913-s006.docx]

| Modules | N | Gene |
| --- | --- | --- |
| Module 1 | 53 | IL6, TNF, AKT1, TP53, ALB, CXCL8, JUN, MAPK3, MAPK8, MAPK1, IL1B, TLR4, CASP3, IL10, PTGS2, MYC, MAPK14, ICAM1, IFNG, HMOX1, FN1, RELA, FOS, CAT, CYCS, MYD88, CXCL10, CCL5, CXCL1, IL18, TLR3, VCAM1, CXCR4, NFKB1, CASP8, PPARG, MPO, NFKBIA, CASP1, TRAF6, MMP2, SOD2, CD86, HMGB1, CASP9, NOS2, TGFB1, SERPINE1, IRF3, CCL4, MMP3, SMAD2, CTGF |
| Module 2 | 25 | MDM2, CDKN1A, EGR1, MAPK9, IFNB1, MIF, CD83, MCL1, IKBKB, XIAP, CD80, IL1A, FCGR2B, HSP90AA1, NFE2L2, CD274, CCL20, CREB1, DDIT3, LMNA, DUSP1, SQSTM1, ATF3, PARP1, CCL3 |

Supplementary material 6: The details of two modules
